# Suicidal Ideation and Behaviors Among High School Students **—** Youth Risk Behavior Survey, United States, 2019

**DOI:** 10.15585/mmwr.su6901a6

**Published:** 2020-08-21

**Authors:** Asha Z. Ivey-Stephenson, Zewditu Demissie, Alexander E. Crosby, Deborah M. Stone, Elizabeth Gaylor, Natalie Wilkins, Richard Lowry, Margaret Brown

**Affiliations:** ^1^Division of Injury Prevention, National Center for Injury Prevention and Control, CDC; ^2^Division of Adolescent and School Health, National Center for HIV/AIDS, Viral Hepatitis, STD, and TB Prevention, CDC; ^3^Office of the Director, National Center for HIV/AIDS, Viral Hepatitis, STD, and TB Prevention, CDC

## Abstract

Suicide is the second leading cause of death among high school-aged youths 14–18 years after unintentional injuries. This report summarizes data regarding suicidal ideation (i.e., seriously considered suicide) and behaviors (i.e., made a suicide plan, attempted suicide, and made a suicide attempt requiring medical treatment) from CDC’s 2019 Youth Risk Behavior Survey. Results are reported overall and by sex, grade, race/ethnicity, sexual identity, and sex of sexual contacts, overall and within sex groups. Trends in suicide attempts during 2009–2019 are also reported by sex, race/ethnicity, and grade. During 2009–2019, prevalence of suicide attempts increased overall and among female, non-Hispanic white, non-Hispanic black, and 12th-grade students. Data from 2019 reflect substantial differences by demographics regarding suicidal ideation and behaviors. For example, during 2019, a total of 18.8% of students reported having seriously considered suicide, with prevalence estimates highest among females (24.1%); white non-Hispanic students (19.1%); students who reported having sex with persons of the same sex or with both sexes (54.2%); and students who identified as lesbian, gay, or bisexual (46.8%). Among all students, 8.9% reported having attempted suicide, with prevalence estimates highest among females (11.0%); black non-Hispanic students (11.8%); students who reported having sex with persons of the same sex or with both sexes (30.3%); and students who identified as lesbian, gay, or bisexual (23.4%). Comprehensive suicide prevention can address these differences and reduce prevalence of suicidal ideation and behaviors by implementing programs, practices, and policies that prevent suicide (e.g., parenting programs), supporting persons currently at risk (e.g., psychotherapy), preventing reattempts (e.g., emergency department follow-up), and attending to persons who have lost a friend or loved one to suicide.

## Introduction

Suicidal behavior presents a major challenge to public health in the United States and globally (*1*). Although fatal (i.e., suicide) and nonfatal (e.g., suicide attempts) suicidal behaviors are a public health concern across the life span, they are of particular concern for youths and young adults aged 10–24 years. During 2018, a total of 48,344 persons (all ages) died from suicide, and suicide was the 10th leading cause of death overall in the United States, accounting for approximately 1.7% of all deaths (*2*). Among high school–aged youths (14–18 years), 2,039 suicides occurred that year, making it the second leading cause of death for this age group after unintentional injuries (n = 2,590). Suicide accounted for approximately 33.9% or approximately one of every three injury-related deaths among this age group (*2*). During 2009–2018, suicide rates among youths aged 14–18 years increased by 61.7% from 6.0 to 9.7 per 100,000 population (*2*). Although suicide is a major public health problem, many more youths make suicide attempts and struggle with suicidal ideation. For example, during 2018, according to data from a nationally representative sample of emergency departments (EDs), approximately 95,000 youths aged 14–18 years visited EDs for self-harm injuries (*2*).

One objective of the *Healthy People 2020* Mental Health and Mental Disorders is to reduce suicide attempts by adolescents that resulted in an injury, poisoning, or overdose that had to be treated by a doctor or nurse (*3*). The Youth Risk Behavior Survey (YRBS) monitors six categories of priority health behaviors and experiences among adolescents, with four questions specifically related to suicide (*4*). This report summarizes 2019 YRBS data regarding suicidal ideation and behaviors among high school students and presents trends in suicide attempts among this population during 2009–2019. The report is intended for decision-makers, prevention program practitioners, and those who work in youth-serving organizations so that they can identify vulnerable youths and take appropriate action to direct prevention resources to those young persons.

## Methods

### Data Source

This report includes data from the 2009–2019 cycles of the YRBS, a cross-sectional, school-based survey conducted biennially since 1991. Each survey year, CDC collects data from a nationally representative sample of public and private school students in grades 9–12 in the 50 U.S. states and the District of Columbia. Additional information about YRBS sampling, data collection, response rates, and processing is available in the overview report of this supplement (*4*). The overview report also includes information about the classification of sexual identity and sex of sexual contacts and standard data analysis methods. The prevalence estimates for all suicidal ideation and behavior questions for the overall study population and by sex, race/ethnicity, grade, and sexual orientation are available at https://nccd.cdc.gov/youthonline/App/Default.aspx. The full YRBS questionnaire is available at https://www.cdc.gov/healthyyouth/data/yrbs/pdf/2019/2019_YRBS-National-HS-Questionnaire.pdf.

### Measures

Four suicidal ideation and behavior variables are included in this report. Suicidal ideation was measured with the question, “During the past 12 months, did you ever seriously consider attempting suicide?” Making a suicide plan was measured with the question, “During the past 12 months, did you make a plan about how you would attempt suicide?” (These two questions had “yes” or “no” response options.) Suicide attempts were measured with the question, “During the past 12 months, how many times did you actually attempt suicide?” Suicide attempts were assessed by frequency of attempts, but the variable was dichotomized into yes or no responses for analytic purposes. Lastly, students were asked, “If you attempted suicide during the past 12 months, did any attempt result in an injury, poisoning, or overdose that had to be treated by a doctor or nurse?” This question is referred to in this report as, “made a suicide attempt requiring medical treatment.” The response options for the last question were, “I did not attempt suicide during the past 12 months,” “yes,” or “no”; however, this variable was also dichotomized into yes or no responses for analysis.

### Analysis

Analyses of these suicidal ideation and behavior variables included examining associations between each item and demographic characteristics, including sex (male/female), race/ethnicity (non-Hispanic white [white], non-Hispanic black [black], or Hispanic), grade (9, 10, 11, or 12), sexual identity (heterosexual; lesbian, gay, or bisexual [LGB]; or not sure), and sex of sexual contacts (sexual contact with only the opposite sex, sexual contact with only the same sex or both sexes, or no sexual contact). Associations by race/ethnicity, grade, sexual identity, and sex of sexual contacts were calculated for the overall study population but also separately for male and female students. Statistical differences were determined by using chi-square analyses at the p <0.05 level of significance. Linear trends for 2009–2019 were examined for attempted suicide by sex, race/ethnicity, and grade. All analyses of suicidal ideation and behaviors were conducted among the full sample, and analysis of behavior variables was not limited to students who reported suicidal ideation (i.e., analysis conducted among the full sample). Additional information about the methods used to conduct YRBS trend analyses are provided in the overview report of this supplement (*4*).

## Results

### Suicidal Ideation and Behaviors, Overall and by Sex

During the 12 months before the survey, 18.8% of students nationwide reported seriously considered attempting suicide (prevalence significantly higher among female [24.1%] than male [13.3%] students), and among students nationwide, 15.7% of students had made a plan about how they would attempt suicide (prevalence significantly higher among female [19.9%] than male [11.3%] students), and 8.9% of students had attempted suicide ≥1 time (prevalence significantly higher among female [11.0%] than male [6.6%] students) (Table 1). Nationwide, 2.5% of students had made a suicide attempt requiring medical treatment, with a prevalence significantly higher among female (3.3%) than male (1.7%) students.

**TABLE 1:** Percentage of high school students who had seriously considered attempting suicide, had made a suicide plan, had attempted suicide, or had made a suicide attempt requiring medical treatment during the 12 months before the survey, by sex — Youth Risk Behavior Survey, United States, 2019

Behavior	Female % (95% CI)	Male % (95% CI)	Total % (95% CI)	Chi-square (p value)
**Seriously considered attempting suicide**				97.922 (<0.001)
Yes	24.1 (22.3‒26.0)	13.3 (12.2‒14.5)	**18.8 (17.6‒20.0)**	NA
No	75.9 (74.0‒77.7)	86.7 (85.5‒87.8)	**81.2 (80.0‒82.4)**	NA
**Made a suicide plan**				109.568 (<0.001)
Yes	19.9 (18.4‒21.6)	11.3 (10.3‒12.4)	**15.7 (14.6‒16.9)**	NA
No	80.1 (78.4‒81.6)	88.7 (87.6‒89.7)	**84.3 (83.1‒85.4)**	NA
**Attempted suicide**				27.037 (<0.001)
Yes	11.0 (9.7‒12.5)	6.6 (5.5‒8.1)	**8.9 (7.9‒10.0)**	NA
No	89.0 (87.5‒90.3)	93.4 (91.9‒94.5)	**91.1 (90.0‒92.1)**	NA
Made a suicide attempt requiring medical treatment*				10.313 (0.003)
Yes	3.3 (2.6‒4.2)	1.7 (1.3‒2.3)	**2.5 (2.1‒3.0)**	NA
No	96.7 (95.8‒97.4)	98.3 (97.7‒98.7)	**97.5 (97.0‒97.9)**	NA

**Abbreviations:** CI = confidence interval; NA = not applicable.

* Made a suicide attempt that resulted in an injury, poisoning, or overdose that had to be treated by a doctor or nurse.

### Suicidal Ideation and Behaviors by Race/Ethnicity and Grade, Overall and by Sex

Overall, a significant difference occurred in having seriously considered attempting suicide by race/ethnicity (white: 19.1%; black: 16.9%; Hispanic: 17.2%) (Table 2), with a significant difference by race/ethnicity among male students (white: 13.8%; black: 10.7%; Hispanic: 11.4%) but not among female students. No significant differences (overall or by sex) occurred in having seriously considered attempting suicide by grade.

**TABLE 2:** Percentage of high school students who had seriously considered attempting suicide, had made a suicide plan, had attempted suicide, or had made a suicide attempt requiring medical treatment during the 12 months before the survey, by sex, race/ethnicity, and grade — Youth Risk Behavior Survey, United States, 2019

Behavior	Female % (95% CI)	Chi-square (p value)	Male % (95% CI)	Chi-square (p value)	Total % (95% CI)	Chi-square (p value)
**Seriously considered attempting suicide**						
Race/Ethnicity	—*	1.504 (0.230)	—	4.989 (0.005)	**—**	5.870 (0.002)
White, non-Hispanic	24.3 (21.9‒26.9)	—	13.8 (12.3‒15.3)	—	**19.1(17.6‒20.8)**	—
Black, non-Hispanic	23.7 (20.7‒27.1)	—	10.7 (8.2‒13.7)	—	**16.9 (15.3‒18.7)**	—
Hispanic	22.7 (19.3‒26.5)	—	11.4 (9.8‒13.3)	—	**17.2 (15.2‒19.4)**	—
Grade	—	0.209 (0.889)	—	0.790 (0.507)	**—**	0.820 (0.491)
9	23.7 (20.7‒27.0)	—	11.9 (9.9‒14.2)	—	**17.7 (15.7‒19.8)**	—
10	23.6 (20.3‒27.3)	—	13.2 (11.1‒15.8)	—	**18.5 (16.1‒21.1)**	—
11	24.9 (22.5‒27.6)	—	13.6 (11.5‒16.0)	—	**19.3 (17.7‒21.1)**	—
12	24.0 (20.7‒27.6)	—	14.9 (12.4‒17.7)	—	**19.6 (17.5‒21.9)**	—
**Made a suicide plan**						
Race/Ethnicity	—	1.652 (0.194)	—	2.358 (0.087)	—	3.043 (0.041)
White, non-Hispanic	19.2 (16.9‒21.8)	—	12.0 (10.6‒13.5)	—	**15.7 (14.1‒17.4)**	—
Black, non-Hispanic	20.4 (17.6‒23.5)	—	10.1 (7.3‒13.9)	—	**15.0 (12.9‒17.5)**	—
Hispanic	19.6 (16.9‒22.6)	—	9.6 (8.0‒11.4)	—	**14.7 (13.0‒16.7)**	—
Grade	—	0.461 (0.711)	—	3.195 (0.035)	**—**	0.652 (0.587)
9	20.4 (17.9‒23.2)	—	9.5 (7.9‒11.4)	—	**14.8 (13.1‒16.6)**	—
10	20.3 (17.2‒23.7)	—	10.4 (8.6‒12.4)	—	**15.4 (13.4‒17.7)**	—
11	20.4 (17.6‒23.5)	—	12.1 (10.3‒14.2)	—	**16.4 (14.5‒18.5)**	—
12	18.5 (15.7‒21.6)	—	13.6 (11.4‒16.1)	—	**16.2 (14.3‒18.3)**	—
**Attempted suicide**						
Race/Ethnicity	—	2.973 (0.044)	—	1.505 (0.229)	**—**	2.866 (0.050)
White, non-Hispanic	9.4 (7.8‒11.3)	—	6.4 (5.1‒7.8)	—	**7.9 (6.9‒9.1)**	—
Black, non-Hispanic	15.2 (10.8‒20.9)	—	8.5 (5.6‒12.9)	—	**11.8 (8.7‒15.9)**	—
Hispanic	11.9 (9.0‒15.6)	—	5.5 (3.9‒7.6)	—	**8.9 (7.1‒11.1)**	—
Grade	—	1.878 (0.150)	—	0.384 (0.765)	**—**	0.311 (0.817)
9	12.8 (10.7‒15.3)	—	6.0 (4.5‒7.9)	—	**9.4 (7.9‒11.1)**	—
10	11.0 (9.1‒13.3)	—	6.5 (4.7‒9.0)	—	**8.8 (7.4‒10.5)**	—
11	10.4 (8.1‒13.3)	—	6.7 (5.2‒8.8)	—	**8.6 (7.1‒10.4)**	—
12	9.4 (6.9‒12.6)	—	7.3 (5.2‒10.0)	—	**8.5 (6.8‒10.6)**	—
**Made a suicide attempt requiring medical treatment^†^**						
Race/Ethnicity	—	0.446 (0.721)	—	1.583 (0.210)	**—**	1.387 (0.262)
White, non-Hispanic	2.9 (1.9‒4.4)	—	1.2 (0.8‒1.9)	—	**2.1 (1.5‒2.8)**	—
Black, non-Hispanic	3.8 (2.3‒6.2)	—	2.9 (1.5‒5.5)	—	**3.3 (2.2‒4.9)**	—
Hispanic	3.6 (2.6‒4.9)	—	2.3 (1.4‒3.9)	—	**3.0 (2.3‒3.8)**	—
Grade	—	0.406 (0.750)	—	0.571 (0.638)	**—**	0.274 (0.844)
9	3.3 (2.3‒4.8)	—	1.3 (0.7‒2.3)	—	**2.3 (1.7‒3.1)**	—
10	3.6 (2.3‒5.5)	—	1.7 (0.9‒3.3)	—	**2.7 (1.8‒3.9)**	—
11	2.7 (1.7‒4.3)	—	2.0 (1.2‒3.2)	—	**2.3 (1.7‒3.3)**	—
12	3.4 (2.2‒5.3)	—	1.9 (1.0‒3.9)	—	**2.7 (2.0‒3.7)**	—

**Abbreviation:** CI = Confidence interval.

*Not applicable.

^†^ Made a suicide attempt that resulted in an injury, poisoning, or overdose that had to be treated by a doctor or nurse.

Among students reporting having made a suicide plan, a significant difference occurred by race and ethnicity overall (white: 15.7%; black: 15.0%; Hispanic: 14.7%) but not among male or female students. No significant difference occurred in having made a suicide plan by grade overall or among female students, but a significant difference was identified among male students (9th grade: 9.5%; 10th grade: 10.4%; 11th grade: 12.1%; 12th grade: 13.6%). Students who had attempted suicide were significantly different by race/ethnicity overall (white: 7.9%; black: 11.8%; Hispanic: 8.9%) and among female students (white: 9.4%; black: 15.2%; Hispanic: 11.9%) but not among male students. No significant differences existed in having attempted suicide by grade (overall or by sex). In addition, no significant difference in having made a suicide attempt requiring medical treatment was noted by race/ethnicity or grade, overall or by sex.

### Suicidal Ideation and Behaviors by Sexual Identity and Sex of Sexual Contacts, Overall and by Sex

A significant difference occurred in having seriously considered attempting suicide by sexual identity overall (heterosexual: 14.5%; LGB: 46.8%; not sure: 30.4%) (Table 3) and among both female (heterosexual: 18.0%; LGB: 49.0%; not sure: 35.9%) and male (heterosexual: 11.4%; LGB: 40.4%; not sure: 21.7%) students. Similarly, having seriously considered attempting suicide varied by sex of sexual contacts, overall (had sexual contact with only the opposite sex: 19.4%; had sexual contact with only the same sex or both sexes: 54.2%; had no sexual contact: 13.0%), among female (had sexual contact with only the opposite sex: 25.3%; had sexual contact with only the same sex or both sexes: 59.2%; had no sexual contact: 16.2%), and among male (had sexual contact with only the opposite sex: 14.6%; had sexual contact with only the same sex or both sexes: 39.1%; had no sexual contact: 9.7%) students.

**TABLE 3:** Percentage of high school students who had seriously considered attempting suicide, had made a suicide plan, had attempted suicide, or had made a suicide attempt requiring medical treatment during the 12 months before the survey, by sex, sexual identity, and sex of sexual contacts — Youth Risk Behavior Survey, United States, 2019

Behavior	Female % (95% CI)	Chi-square (p value)	Male % (95% CI)	Chi-square (p value)	Total % (95% CI)	Chi-square (p value)
**Seriously considered attempting suicide**						
Sexual identity	—	75.728 (<0.001)	—	22.231 (<0.001)	—	88.194 (<0.001)
Heterosexual	18.0 (16.3‒20.0)	—*	11.4 (10.4‒12.6)	—	14.5 (13.4‒15.7)	—
LGB	49.0 (44.8‒53.3)	—	40.4 (33.9‒47.1)	—	46.8 (43.1‒50.6)	—
Not sure	35.9 (29.5‒42.9)	—	21.7 (14.8‒30.5)	—	30.4 (25.4‒35.9)	—
Sex of sexual contacts	—	64.007 (<0.001)	—	13.972 (<0.001)	—	66.938 (<0.001)
Opposite sex only	25.3 (22.8‒28.0)	—	14.6 (12.9‒16.5)	—	19.4 (17.6‒21.4)	—
Same sex only or both sexes	59.2 (52.5‒65.6)	—	39.1 (29.3‒49.9)	—	54.2 (49.0‒59.3)	—
No sexual contact	16.2 (14.2‒18.3)	—	9.7 (8.1‒11.7)	—	13.0 (11.8‒14.3)	—
**Made a suicide plan**						
Sexual identity	—	66.568 (<0.001)	—	19.732 (<0.001)	—	90.368 (<0.001)
Heterosexual	14.6 (13.2‒16.0)	—	9.9 (8.9‒11.0)	—	12.1 (11.1‒13.1)	—
LGB	42.4 (38.4‒46.4)	—	33.0 (26.4‒40.3)	—	40.2 (36.6‒44.0)	—
Not sure	28.1 (22.1‒35.0)	—	17.4 (11.8‒24.8)	—	23.9 (19.4‒29.0)	—
Sex of sexual contacts	—	56.442 (<0.001)	—	18.435 (<0.001)	—	62.470 (< 0.001)
Opposite sex only	20.7 (18.4‒23.3)	—	12.9 (11.5‒14.6)	—	16.5 (14.9‒18.1)	—
Same sex only or both sexes	48.2 (42.8‒53.6)	—	31.2 (23.8‒39.7)	—	44.0 (39.7‒48.4)	—
No sexual contact	13.8 (12.3‒15.6)	—	7.9 (6.7‒9.4)	—	10.9 (9.8‒12.1)	—
**Attempted suicide**						
Sexual identity	—	26.919 (<0.001)	—	15.972 (<0.001)	—	40.352 (<0.001)
Heterosexual	7.9 (6.6‒9.4)	—	5.1 (4.2‒6.3)	—	6.4 (5.6‒7.4)	—
LGB	23.6 (20.0‒27.6)	—	23.8 (17.8‒31.1)	—	23.4 (20.0‒27.1)	—
Not sure	15.2 (9.6‒23.3)	—	16.4 (9.9‒26.0)	—	16.1 (11.1‒22.8)	—
Sex of sexual contacts	—	58.123 (<0.001)	—	12.379 (<0.001)	—	66.202 (<0.001)
Opposite sex only	11.4 (9.5‒13.5)	—	7.5 (5.8‒9.6)	—	9.3 (7.9‒10.8)	—
Same sex only or both sexes	31.4 (27.0‒36.1)	—	26.5 (17.5‒38.0)	—	30.3 (25.9‒35.0)	—
No sexual contact	6.1 (4.8‒7.8)	—	3.5 (2.6‒4.8)	—	4.8 (4.0‒5.8)	—
**Made a suicide attempt requiring medical treatment^†^**						
Sexual identity	—	7.893 (0.001)	—	5.592 (0.008)	—	13.034 (<0.001)
Heterosexual	2.3 (1.6‒3.2)	—	1.3 (0.9‒1.9)	—	1.7 (1.4‒2.2)	—
LGB	6.6 (5.0‒8.7)	—	5.9 (3.2‒10.6)	—	6.3 (4.8‒8.3)	—
Not sure	3.8 (1.6‒8.4)	—	7.6 (3.6‒15.2)	—	5.2 (3.0‒9.0)	—
Sex of sexual contacts	—	14.728 (<0.001)	—	10.517 (<0.001)	—	23.046 (<0.001)
Opposite sex only	3.4 (2.4‒4.8)	—	1.9 (1.3‒2.9)	—	2.6 (2.0‒3.3)	—
Same sex only or both sexes	10.4 (7.5‒14.2)	—	9.4 (4.9‒17.6)	—	10.2 (7.6‒13.4)	—
No sexual contact	1.4 (0.8‒2.4)	—	0.5 (0.3‒1.1)	—	1.0 (0.6‒1.5)	—

**Abbreviations:** CI = confidence interval; LGB = lesbian, gay, or bisexual.

*Not applicable.

^†^ Made a suicide attempt that resulted in an injury, poisoning, or overdose that had to be treated by a doctor or nurse.

Overall, a significant difference occurred in having made a suicide plan by sexual identity (heterosexual: 12.1%; LGB: 40.2%; not sure: 23.9%), with a significant difference among both female (heterosexual: 14.6%; LGB: 42.4%; not sure: 28.1%) and male (heterosexual: 9.9%; LGB: 33.0%; not sure: 17.4%) students. Similarly, a significant difference was noted among students having made a suicide plan by sex of sexual contacts, overall (had sexual contact with only the opposite sex: 16.5%; had sexual contact with only the same sex or both sexes: 44.0%; had no sexual contact: 10.9%), with a significant difference among both female (had sexual contact with only the opposite sex: 20.7%; had sexual contact with only the same sex or both sexes: 48.2%; had no sexual contact: 13.8%) and male (had sexual contact with only the opposite sex: 12.9%; had sexual contact with only the same sex or both sexes: 31.2%; had no sexual contact: 7.9%) students.

A significant difference existed in having attempted suicide by sexual identity, overall (heterosexual: 6.4%; LGB: 23.4%; not sure: 16.1%) and among both female (heterosexual: 7.9%; LGB: 23.6%; not sure: 15.2%) and male (heterosexual: 5.1%; LGB: 23.8%; not sure: 16.4%) students. Similarly, a significant difference was identified in having attempted suicide by sex of sexual contacts, overall (had sexual contact with only the opposite sex: 9.3%; had sexual contact with only the same sex or both sexes: 30.3%; no sexual contact: 4.8%), with a significant difference among both female (had sexual contact with only the opposite sex: 11.4%; had sexual contact with only the same sex or both sexes: 31.4%; no sexual contact: 6.1%) and male (had sexual contact with only the opposite sex: 7.5%; had sexual contact with only the same sex or both sexes: 26.5%; no sexual contact: 3.5%) students.

Finally, a significant difference occurred in having made a suicide attempt requiring medical treatment by sexual identity, overall (heterosexual: 1.7%; LGB: 6.3%; not sure: 5.2%) and among both female (heterosexual: 2.3%; LGB: 6.6%; not sure: 3.8%) and male (heterosexual: 1.3%; LGB: 5.9%; not sure: 7.6%) students. A significant difference also was noted in having made a suicide attempt requiring medical treatment by sex of sexual contacts, overall (had sexual contact with only the opposite sex: 2.6%; had sexual contact with only the same sex or both sexes: 10.2%; had no sexual contact: 1.0%) and among both female (had sexual contact with only the opposite sex: 3.4%; had sexual contact with only the same sex or both sexes: 10.4%; had no sexual contact: 1.4%) and male (had sexual contact with only the opposite sex: 1.9%; had sexual contact with only the same sex or both sexes: 9.4%; had no sexual contact: 0.5%) students.

### Trends in Suicide Attempts, Overall and by Sex, Race/Ethnicity, and Grade 

**FIGURE 1:**
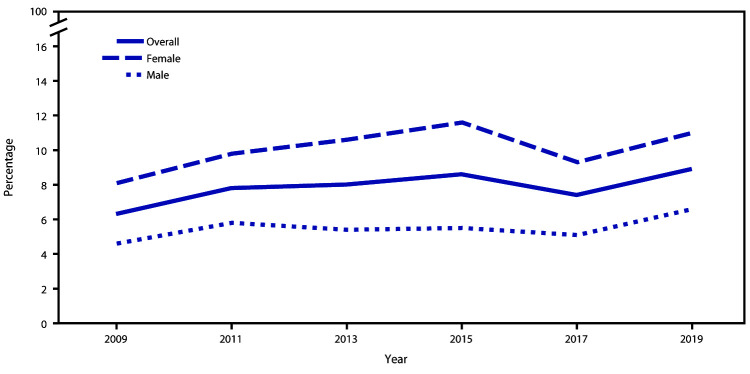
Percentage of high school students who attempted suicide during the 12 months before the survey, overall and by sex — Youth Risk Behavior Survey, United States, 2009**‒**2019

**FIGURE 2:**
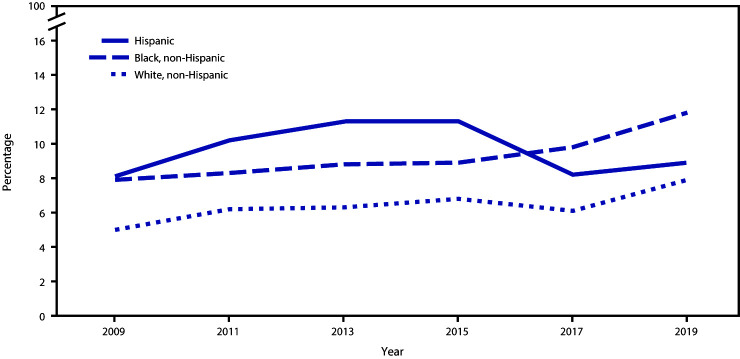
Percentage of high school students who attempted suicide during the 12 months before the survey, by race/ethnicity — Youth Risk Behavior Survey, United States, 2009**‒**2019

**FIGURE 3:**
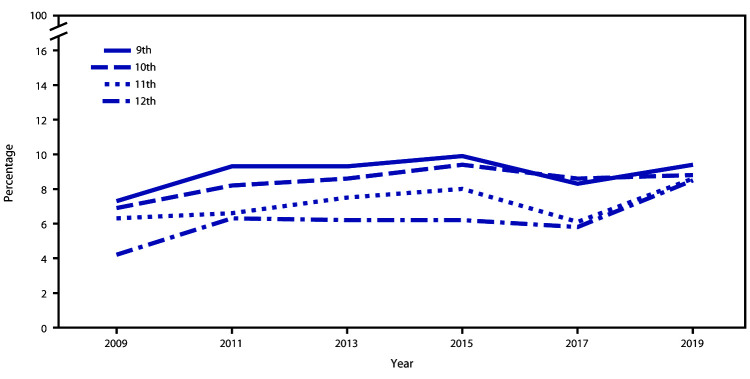
Percentage of high school students who attempted suicide during the 12 months before the survey, by grade — Youth Risk Behavior Survey, United States, 2009**‒**2019

Among the total student population, the percentage of students who had attempted suicide ≥1 time during the 12 months before the survey experienced a significant linear increase from 6.3% during 2009 to 8.9% during 2019 (Figures 1, 2, and 3). Among female students, a significant linear increase (from 8.1% to 11.0%) occurred in the prevalence of having attempted suicide. No significant linear change was observed for the prevalence of having attempted suicide among male students. By race/ethnicity, significant linear increases in having attempted suicide were observed for white (from 5.0% to 7.9%) and black (from 7.9% to 11.8%) but not Hispanic students. By grade, a significant linear increase in having attempted suicide was observed only for 12th-grade students (from 4.2% to 8.5%).

## Discussion

During 2019, approximately one in five (18.8%) youths had seriously considered attempting suicide, one in six (15.7%) had made a suicide plan, one in 11 (8.9%) had made an attempt, and one in 40 (2.5%) had made a suicide attempt requiring medical treatment. Linear trends in suicide attempts have increased during 2009–2019 overall and among certain demographic groups.

The 2019 YRBS data highlight considerable differences in suicidal ideation, plans, attempts, and attempts requiring medical treatment. Consistent with previous research, during 2019, females had more suicidal ideation, suicide plans, and suicide attempts, including attempts requiring medical treatment, than males (*5*). Certain racial/ethnic differences also were identified. For example, black male students had the lowest prevalence estimates of suicidal ideation. Regarding suicide attempts, black students (male and female) had the highest prevalence estimates. This finding is consistent with previous research (*6*). Also consistent with previous research are the study findings regarding sexual orientation and sex of sexual contacts (*5*). Namely, prevalence estimates of suicidal ideation, suicide plans, attempts, and attempts requiring medical treatment were highest among sexual minority youths, those who identified as LGB, and youths who reported having had sexual contact with the same or with both sexes during 2019.

Adolescence is a developmental stage often characterized by rapid and extensive physical and psychosocial changes (*7*). It also represents a time for expanded identity development, with sexual identity development representing a complex, multidimensional, and often stressful process for youths (*8*). The potential dissonance between sexual identity and behavior and the social rejection sexual minority youths often experience can contribute to increased suicidal ideation and behaviors along with an increased risk for suicide (*9**,**10*). Because of the high prevalence of suicidal ideation and behaviors among sexual minority youths, additional research is needed to determine how best to support this vulnerable group. Such research might evaluate strategies designed to reduce sexual minority stress (e.g., discrimination and victimization resulting from sharing one’s sexual orientation) (*11*) and unhealthy behaviors (e.g., substance use) and the resultant impact on suicidal ideation and behaviors.

Suicide attempts are a known risk factor for and the greatest predictor of death by suicide (*12*), which is the rationale for investigating trends only on this outcome. The number of children and adolescents who sought medical treatment at EDs for suicide attempts increased sharply from 2007 (540,000) to 2015 (960,000) (*13*). Either a linear increase or no change in suicide attempts by variables reported here (i.e., sex, race/ethnicity, and grade) was identified for 2009–2019. Although for a different period (1991–2017), other researchers also have reported that suicide attempts among black students increased significantly (*6*). More specifically, previous findings indicated that suicide attempts increased at an accelerating rate among black females, and black male youths had a substantial increase in attempts requiring medical treatment during the period (*6*). Future studies are needed to continue monitoring trends in suicidal ideation and behavior for black students and other race/ethnicity groups. For example, such studies might include more detailed analyses among the American Indian/Alaska Native youth population who have been reported to be at increased risk for suicidal behaviors (*6*).

In this analysis, one notable finding emerged by sex and grade; a substantial increase in making a suicide plan occurred among males as grade increased. To address this trend, schools can consider a sex-by-grade–specific approach to implementing suicide prevention or intervention activities.

## Limitations

General limitations for the YRBS are available in the overview report of this supplement (*4*). The findings in this report are subject to at least one additional limitation. This analysis is conducted among all students (i.e., does not separate ideation from behaviors); suicide patterns might differ between those who experienced suicidal ideation and those who did not.

## Future Directions

To address the health differences in suicidal ideation and behaviors observed by student demographics and to decrease these outcomes overall, a comprehensive approach to suicide prevention, including programs, practices, and policies based on the best available evidence, is needed. Such an approach addresses the range of risk and protective factors occurring across the individual, relationship, community, and societal levels. A comprehensive approach also seeks to prevent suicide risk, identify and support youths at increased risk, prevent attempts and reattempts, and help survivors of suicide loss (i.e., those grieving the death of a friend or loved one). States and communities, including school communities, can use strategies with such best available evidence as that documented in the CDC Preventing Suicide Technical Package (*14*).

Preventing adverse childhood experiences (e.g., child maltreatment) can help reduce suicide risk among adolescents through strategies that promote safe, stable, nurturing relationships and environments in childhood (*15*). Other strategies in a comprehensive approach to suicide prevention include supporting families by strengthening economic supports and teaching coping and problem-solving skills among children, adolescents, and their parents; promoting connectedness between youths and their schools, teachers, peers, and family; creating protective environments in schools and at home (e.g., limiting access to such lethal means among students at risk as medications and firearms); promoting help-seeking behaviors; reducing stigma; and training teachers and adults in recognizing signs of suicide and responding effectively through referrals to evidence-based treatment (e.g., cognitive-behavioral therapy) (*14*). Finally, schools and the media should respond to and report on suicides in ways that are supportive and responsible (e.g., not sensationalizing deaths), thereby avoiding additional suicides (i.e., suicide contagion) (*14*).

## Conclusion

Suicide is a leading cause of death among youths; however, many more youths are at risk for suicide as a result of experiencing suicidal ideation, making suicide plans, and attempting suicide, making a focus on nonfatal suicidal behavior a crucial public health priority. During 2009–2019, trends in suicide attempts among adolescents increased overall and among many demographic groups. Prevalence estimates of suicidal ideation, suicide plans, attempts, and attempts requiring medical treatment were highest among sexual minority youths and youths who reported having had sexual contact with the same or with both sexes. Regarding differences by race/ethnicity, black students had the highest prevalence estimates for attempted suicide. Factors at the individual, relationship, community, and societal levels likely contribute to the differences in suicide attempts among different racial/ethnic groups and sexual minority youths and the differences observed by sex and grade. More research is needed to better understand the risk and protective factors to determine which suicide prevention strategies might best serve each group. The findings in this report underscore the importance of a comprehensive approach to suicide prevention, which would provide necessary support to those at risk, decrease suicidal ideation and behaviors, and ultimately prevent suicide among youths and save lives.
